# Advances and Clinical Trials Update in the Treatment of Diffuse Intrinsic Pontine Gliomas

**DOI:** 10.1159/000529099

**Published:** 2023-01-13

**Authors:** Cecilia Dalle Ore, Christina Coleman, Nalin Gupta, Sabine Mueller

**Affiliations:** ^a^Department of Neurological Surgery, University of California San Francisco, San Francisco, California, USA; ^b^Division of Hematology/Oncology, Montreal Children's Hospital, McGill University Health Centre, Montreal, Québec, Canada; ^c^Department of Pediatrics, University of California San Francisco, San Francisco, California, USA; ^d^Department of Neurology, University of California San Francisco, San Francisco, California, USA

**Keywords:** Diffuse intrinsic pontine gliomas, Biopsy, Immunotherapy, Convection-enhanced delivery

## Abstract

**Background:**

Diffuse intrinsic pontine gliomas (DIPGs) are high-grade gliomas (HGGs) that occur primarily in children, and represent a leading cause of death in pediatric patients with brain tumors with a median overall survival of only 8–11 months.

**Summary:**

While these lesions were previously thought to behave similarly to adult HGG, emerging data have demonstrated that DIPG is a biologically distinct entity from adult HGG frequently driven by mutations in the histone genes H3.3 and H3.1 not found in adult glioma. While biopsy of DIPG was historically felt to confer unacceptable risk of morbidity and mortality, multiple studies have demonstrated that stereotactic biopsy of DIPG is safe, allowing not only for improved understanding of DIPG but also forming the basis for protocols for personalized medicine in DIPG. However, current options for personalized medicine in DIPG are limited by the lack of efficacious targeted therapies for the mutations commonly found in DIPG. Multiple treatment modalities including targeted therapies, immunotherapy, convection-enhanced delivery, and focused ultrasound are in various stages of investigation.

**Key Message:**

Increasing frequency of biopsy for DIPG has identified distinct driving mutations that may serve as therapeutic targets. Novel treatment modalities are under investigation.

## Introduction

Diffuse intrinsic pontine gliomas (DIPGs) are high-grade gliomas (HGGs) which occur primarily in children. They belong to the category of diffuse midline glioma (DMG), as defined in the current WHO classification, which occur in midline locations and share similar genetic changes and clinical behavior. DIPG/DMG represents 10–15% of all pediatric brain tumors and is a leading cause of death in children with brain tumors [1, 2]. The median overall survival in patients with DIPG is only 8–11 months, and survival at 2 years is <10% [2]. DIPG was historically a clinical and radiographic diagnosis, with diagnosis based on presentation with cranial neuropathies and/or pyramidal symptoms with an expansile, diffuse pontine mass; most patients were diagnosed and treated without tissue confirmation [3]. Based on histology of samples derived at autopsy and an aggressive disease course, DIPG was assumed to be biologically similar to glioblastoma and other adult HGG [3]. However, extrapolating adult HGG treatment regimens to children with DIPG has failed despite extensive efforts over the last several decades [1]. The mainstay of glioblastoma treatment in the adult population, temozolomide combined with radiation therapy, does not improve survival in patients with DIPG [1, 4]. Standalone radiation therapy is the traditional standard of care in patients with DIPG, providing transient improvement of neurologic deficits in symptomatic patients, reducing steroid utilization [2], and conferring a 3-month survival benefit [5]. However, further trials have failed to improve overall survival in patients with DIPG.

## Biological Features of DIPG

Attempts at understanding the tumor biology of DIPG were previously limited by the lack of available tissue. Due to its location in the pons, biopsy of these tumors was thought to be associated with unacceptable morbidity [6, 7]. In the last 10–15 years, several groups have demonstrated the feasibility of stereotactic biopsy with a low morbidity and mortality [8, 9]. In single institution retrospective series of 130 DIPG biopsies by Puget et al. [10], they found a 3.9% rate of transient neurologic deficits with no mortality and no permanent deficits and a diagnostic yield was 100%. Reviews and meta-analyses of the literature have since reported similar complication rates of less than 5%, with no mortality in previously published case series and a diagnostic yield of over 96% [11].

**Fig. 1:**
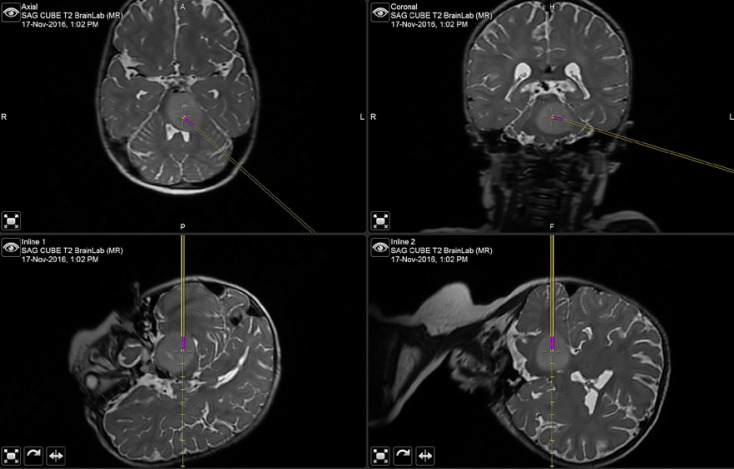
A representative biopsy trajectory used to access an infiltrating pontine glioma. The bottom panels show the actual trajectory through the cerebellum and middle cerebellar peduncle to reach the posterior portion of the mass. The magenta color shows the location of the aperture in the biopsy needle that is used to obtain the tissue core.

Tissue obtained from stereotactic biopsy (Fig. 1), in combination with autopsy specimens, has led to a new understanding of DIPG biology. Early studies demonstrated that DIPG behaves in a biologically distinct fashion from glioblastoma and other adult gliomas. In a review by Jansen et al. [1] of 108 biopsies performed in 26 prospective clinical trials involving 561 children with newly diagnosed DIPG, DIPG was found to represent a range of histologic tumor grades, including 22 WHO grade II gliomas, 40 WHO grade III gliomas, 27 glioblastoma, 15 malignant glioma, and 4 histologically undefined tumors. However, contrary to adult gliomas, WHO grade did not correlate with tumor behavior, overall survival, or progression-free survival [1, 10].

Early data from biopsy studies have further demonstrated that DIPG represents a biologically distinct entity from GBM and other adult glioma. Well-characterized adult glioma mutations including IDH1, BRAF, FGFR1, MYB, and TERT are atypical for DIPG [12, 13, 14]. Instead, DIPGs frequently have mutations in histone genes H3.3 or H3.1 [12], with 45–80% of DIPG harboring either the H3.3K27M or H3.1K27M mutation [12]. Methylation at H3K27 plays a key role in regulating the expression of genes involved in development [15]. The H3K27M mutation results in a global reduction of H3K27 methylation, resulting in reduction of gene expression at these loci [15]. Other mutations include PDGFRA gains or amplifications in 30–36% of patients [16]; ACVR mutations in 20% of patients [12, 17, 18]; FGFR mutations [14]; gain of RTK and RB pathway components including KRAS, AKT1, PIK3CA, CDK4, and CDK5 in 47–69% of patients [18, 19]; increased MET, EGFR, and ERBB1 [19, 20]; and enrichment in P53 mutations [14]. FGFR1, ACVR1, PDGFRA mutations have been reported to be mutually exclusive and associated with H3K27M [14].

These mutations have been also associated with distinct clinical features: K27M H3.1 tumors frequently resemble anaplastic astrocytoma on histopathology and tend to occur in younger patients, K27M H3.3 tumors are associated with TP53 and ATRX mutations and more frequently resemble glioblastoma [12, 14], and molecularly silent DIPG often resembles low-grade glioma on histopathology [12]. However, there is no significant difference in overall survival between these groups [12].

## Personalized Medicine in DIPG

Following the novel identification of multiple mutations in DIPG and an increased understanding of the heterogenous nature of these lesions, trials emerged paired stereotactic biopsy with molecularly targeted therapies. The DIPG Biology and Treatment Study both evaluated the feasibility of stereotactic biopsy at diagnosis for genetic analysis and designation of treatment groups and subsequently treated enrolled patients with FDA-approved therapies based on identified mutations [3]: all patients were treated with radiation therapy and bevacizumab, but patients could also receive erlotinib based on the presence of an EGFR mutation and temozolomide based on MGMT promoter methylation [3]. Similar to prior reports regarding safety and feasibility of biopsy, DIPG-BATS reported no mortality and minimal morbidity in association with biopsy, with one patient who developed left hemiparesis. DIPG-BATS also demonstrated that this workflow could be implemented in a clinically appropriate time period, with all put one patient undergoing radiation therapy within 21 days of biopsy, and sufficient tissue being obtained for genetic studies and treatment stratification in 96% of patients biopsied.

Another early feasibility study, PNOC003, incorporated whole exome sequencing and RNA sequencing of paired tumor and normal tissues in DIPG. Following sequencing, medical treatment utilizing of up to 4 FDA-approved drugs was utilized based on the tumor's genetic profile [21]. PNOC003 again demonstrated the potential utility of personalized medicine in DIPG, with a personalized treatment recommendation based on sequencing results provided within 21 business days of tissue collection [21]. Personalized medicine inherently requires the identification and development of medications that effectively treat identified mutations. Multiple agents are currently under development in an effort to establish additional targeted treatment regimens for mutations harbored in DIPG. Mutations in DIPG are often subclonal and may evolve over time; effective treatment is likely to require combination therapy [5].

## Molecularly Targeted Therapies

Given the high prevalence of histone mutations in DIPG, HDAC inhibitors and other agents that target histone methylation have been an area of intense research and clinical interest. The HDAC inhibitor panobinostat has been shown to be effective in preclinical studies, producing an increase in H3K27 trimethylation, a partial rescue of the global hypomethylation phenotype found in DIPG [22], and ultimately extended survival and reduced tumor xenograft proliferation in a murine model [23]. Case reports have reported a survival benefit of panobinostat in combination with reirradiation in 2 cases, and panobinostat has been evaluated in a phase 1 trial utilizing convection-enhanced delivery (CED) [5].

mTOR inhibitors have also been investigated in an effort to target the increased activation of the PI3K/AKT/mTOR pathway, with dual mTOR inhibitors showing promise in some in vitro and xenograft models [24]. However, clinical efficacy of mTOR inhibitors has been limited, which may reflect poor blood-brain barrier penetrance of current agents [25].

The ACVR1 mutation found in 20% of patient has also been an area of preclinical investigation, with ALK2 inhibitors demonstrating modest preclinical efficacy and an ability to cross the blood-brain barrier [17, 26], and the BMP inhibitor noggin and ACVR1 inhibitor LDN212854 both improving overall survival and reducing tumor proliferation in mice with generated PDGAF./HK3.3/ACVR mutant tumors [17]. However, even in this limited model system, agents were not capable of producing a cytotoxic response, and the response generated was attributed largely to off-target effects [17].

## Immunotherapy

DIPG is associated with a minimal immune infiltrate [5], and DIPG tumor cells and associated macrophages express fewer cytokines than glioblastoma [27, 28]. Despite these challenges, some early data have shown promise, and trials are ongoing in an effort to develop efficacious and safe immunotherapies and in various stages of preclinical and clinical studies. Peptide vaccines for H3K27M on MHC class II have been generated and shown to produce a CD8 and Th1-mediated immune response in a murine model [29]. Preliminary investigations of an autologous dendritic cell vaccine have reported that these generate an immune response [30]. Chheda et al. [31] all generated H3.3K27M-specific TCRs with cytotoxic activity that produced glioma cell death and reduced tumor progression in a murine model. Mount et al. [32] produced CAR T cells targeting GD2 in DIPG and reported near-complete tumor clearance and substantially improved survival with peripheral administration. PD-L1 inhibitors have also been trialed and have been reported to produce a slight increase in overall survival [33]; however, efficacy is likely limited by minimal PD-L1 expression in DIPG [33].

## Convection-Enhanced Delivery

**Fig. 2:**
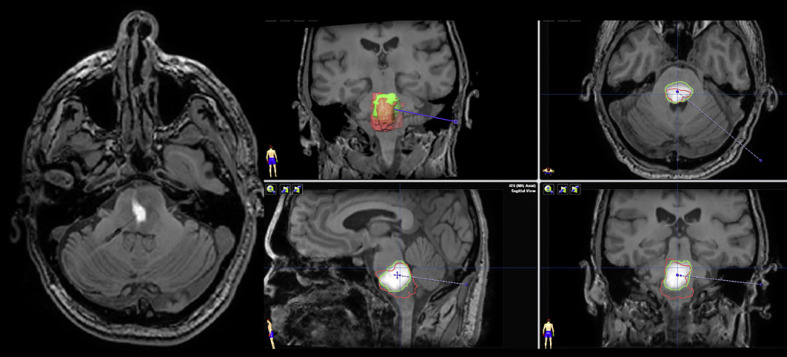
A composite image showing accumulation of gadolinium within a pontine glioma during a CED procedure using an experimental agent. The infusion catheter can be seen in the left panel as a low-signal intensity linear structure. The four panels on the right demonstrate the overall tumor volume in red (as determined from a T2-weighted sequence) and the overlap with the infusion performed (green outline).

Penetration of the blood-brain barrier of agents administered systemically requires molecular weight of <500 Da and high lipophilicity [34]; only 5% of agents meet these parameters [35]. Given that the efficacy of many agents is limited by the blood-brain barrier, authors have proposed utilizing CED to bypass the blood-brain barrier and generate high local concentrations in the area of the tumor while limiting systemic toxicity (Fig. 2). Early studies of CED have demonstrated that CED can safely deliver appropriate medications to the tumor volume, with volumes of distribution of up to 20 cm^3^. CED was notable for producing negligible systemic exposure; in a study by Souweidane et al. utilizing radioimmunotherapy agent, the ratio of lesion-to-whole body radiation absorbed was higher than 1,200. However, concomitant biopsy may not be feasible with CED as it may negatively affect drug distribution, and the logistics of analyzing tissue and establishing a molecularly targeted regimen would therefore potentially require multiple invasive procedures in order to provide molecularly targeted agents via CED.

## Focused Ultrasound

Early preclinical data for the use of focused ultrasound have shown promise in HGG. In a rat model, focused ultrasound in combination with the photosensitizing agent 5-aminolevulinic acid under MR thermometry surveillance produced an increase in survival and decreased tumor growth; 5-aminolevulinic acid and focused ultrasound did not improve survival separately [36]. Focused ultrasound has also been identified as a potential avenue for improved delivery of systemically administered medications in DIPG; in a murine model, the combination of focused ultrasound with IV doxorubicin produced a significantly higher medication concentration within the tumor xenograft and suppressed tumor growth [37]. Clinical trials combining focused ultrasound with systemic medications are underway (NCT05123534).

## Current Trials and Future Directions

A number of trials are ongoing and in development for the treatment of DIPG using a variety of novel approaches and combinations (Table 1). ONC201 is a small molecule dopamine receptor antagonist and mitochondrial protease ClpP caseinolytic protease proteolytic subunit agonist [36]. Initial reports of single agent use of ONC2001 showed that some patients had a survival benefit, particularly those with thalamic tumors [37, 38]. Response in the phase 1 trial was also able to be corelated with CSF cell-free tumor DNA [38]. In a current trial, the use of ONC201 in combination with other targeted therapies is being investigated (NCT05009992). This trial includes a target validation arm as well as correlative studies to help gain understanding of the drugs' mechanism and CNS/tumor penetration. In an effort to overcome drug delivery challenges, the use of MR-guided focused ultrasound is being investigated (NCT05123534). The use of sonodynamic therapy, a combination of drug and ultrasound device, was shown to extend survival in an animal model of glioma [39]. Another exciting strategy for the treatment of DIPG involves harnessing the immune system through the use of genetically altered host T cells. Early results of a phase 1 trial of autologous GD2-CAR T cells in patients with DIPG (NCT04196413) are encouraging [40]. Further trials investigating similar immunotherapeutic approaches are ongoing (NCT04185038, NCT04099797, NCT04196413) and forthcoming (NCT05478837).

## Conflict of Interest Statement

The authors have no conflicts of interest to declare.

## Funding Sources

No authors received funding for this study.

## Author Contributions

Cecilia Dalle Ore performed the initial review and wrote the initial draft of the manuscript. Christina Coleman compiled additional information and contributed to the writing of the manuscript. Nalin Gupta supervised writing of the manuscript and edited the finalized manuscript. Sabine Mueller supervised the design and writing of the finalized manuscript.

## Figures and Tables

**Table 1:** Current and upcoming clinical trials in DIPG

NCT	Title	Biopsy required Y/N	Newly diagnosed or recurrent/progressive disease	Age, years	Type of treatment and mode of delivery
NCT04732065	PNOC023: Open Label Phase 1 and Target Validation Study of ONC206 in Children and Young Adults with Newly Diagnosed or Recurrent Diffuse Midline Glioma (DMG), and Other Recurrent Primary Malignant Central Nervous System (CNS) Tumors	Y	Both	2–21	Oral drug

NCT04773782	A Study of Avapritinib in Pediatric Patients with Solid Tumors Dependent on KIT or PDGFRA Signaling	Y	Recurrent/progressive	2–18	Oral drug

NCT03389802	Phase 1 Study of APX005M in Pediatric CNS Tumors	N	Newly diagnosed	1–21	IV drug (antibody)

NCT03605550	A Phase 1b Study of PTC596 in Children with Newly Diagnosed Diffuse Intrinsic Pontine Glioma and High Grade Glioma	Y	Newly diagnosed	1–21	Oral drug

NCT05009992	A Combination Therapy Trial using an Adaptive Platform Design for Children and Young Adults with Diffuse Midline Gliomas (DMGs) Including Diffuse Intrinsic Pontine Gliomas (DIPGs) at Initial Diagnosis, Post-Radiation Therapy and at Time of Progression	Y	Both	2–39	Oral drug combination

NCT05099003	A Study of the Drug Selinexor with Radiation Therapy in Patients with Newly-iagnosed Diffuse Intrinsic Pontine (DIPG) Glioma and High-Grade Glioma (HGG)	N	Newly diagnosed	1–21	Oral drug

NCT02644460	Abemaciclib in Children with Newly Diagnosed Diffuse Intrinsic Pontine Glioma, and in Children with Recurrent and Refractory Solid Tumors Including Malignant Brain Tumors	Y	Newly diagnosed	2–25	Oral drug

NCT05123534	A Phase 1/2 Study of Sonodynamic Therapy Using SONALA-001 and Exablate 4,000 Type 2 in Patients With DIPG	N	Newly diagnosed	5+	Focused ultrasound device and IV drug

NCT04837547	Peach Trial - Precision Medicine and Adoptive Cellular Therapy for the Treatment of Recurrent Neuroblastoma and Newly Diagnosed Diffuse Intrinsic Pontine Glioma (DIPG)	Y	Newly diagnosed	3–30	Immunotherapy

NCT02992015	Gemcitabine in Newly-Diagnosed Diffuse Intrinsic Pontine Glioma	Y	Newly diagnosed	3–17	IV chemotherapy

NCT04049669	Pediatric Trial of Indoximod with Chemotherapy and Radiation for Relapsed Brain Tumors or Newly Diagnosed DIPG	N	Newly diagnosed	3–21	Oral chemo-immunotherapy

NCT04771897	A Phase 1 Open Label, Multi-Center Study to Evaluate the Safety and Tolerability of BXQ-350 in Children with Newly Diagnosed Diffuse Intrinsic Pontine Glioma (DIPG) and Diffuse Midline Glioma (DMG)	N	Newly diagnosed	1–30	IV drug

NCT04196413	GD2 CAR T Cells in Diffuse Intrinsic Pontine Gliomas (DIPG) & Spinal Diffuse Midline Glioma(DMG)	Y	Both	2–30	Intraventricular immunotherapy

NCT04185038	Study of B7-H3-Specific CAR T Cell Locoregional Immunotherapy for Diffuse Intrinsic Pontine Glioma/Diffuse Midline Glioma and Recurrent or Refractory Pediatric Central Nervous System Tumors	N	Both	1–26	Intraventricular immunotherapy

NCT04099797	C7R-GD2.CAR T Cells for Patients with GD2-Expressing Brain Tumors (GAIL-B)	Y	Both	1–21	Intraventricular immunotherapy

NCT05478837	PNOC018 A Phase 1 Clinical Trial of Autologous T Cells Expressing a TCR Specific for H3.3K27M with Inhibition of Endogenous TCR (KIND T Cells) in HLA-A*0201-Positive Participants with Newly Diagnosed H3.3K27M-Positive Diffuse Midline Gliomas	Y	Newly diagnosed	3–21	IV immunotherapy

NCT03396575	BRAVO: Newly-Diagnosed Brain Stem Gliomas Treated with Adoptive Cellular Therapy during Recovery from Focal Radiotherapy Alone or Focal Radiotherapy and Dose-Intensified Temozolomide (Phase 1)	Y	Newly diagnosed	3–30	Vaccine/immunotherapy

NCT04943848	rFISC-DIPGVax Plus Checkpoint Blockade for the Treatment of Newly Diagnosed DIPG and DMG	N	Newly diagnosed	1–18	Vaccine/immunotherapy

NCT05096481	PEP-CMV Vaccine Targeting CMV Antigen to Treat Newly Diagnosed Pediatric FIGG and DIPG and Recurrent Medulloblastoma	N	Newly diagnosed	3–25	Vaccine
